# Current’s Fluctuations through Molecular Wires Composed of Thiophene Rings

**DOI:** 10.3390/molecules23040881

**Published:** 2018-04-11

**Authors:** Judith Helena Ojeda Silva, Juan Camilo Cortés Peñaranda, Jovanny A. Gómez Castaño, Carlos Alberto Duque

**Affiliations:** 1 Grupo de Física de Materiales, Facultad de Ciencias, Universidad Pedagógica y Tecnológica de Colombia, Tunja, Boyacá 050030, Colombia; juancamilo.cortes@uptc.edu.co; 2 Laboratorio de Química Teórica y Computacional, Grupo de Investigación Química-Física Molecular y Modelamiento Computacional (QUIMOL), Facultad de Ciencias, Universidad Pedagógica y Tecnológica de Colombia, Tunja, Boyacá 050030, Colombia; jovanny.gomez@uptc.edu.co; 3 Grupo de Materia Condensada-UdeA, Instituto de Física, Facultad de Ciencias Exactas y Naturales, Universidad de Antioquia UdeA, Calle 70 No. 52-21, Medellín 050030, Colombia; cduque_echeverri@yahoo.es

**Keywords:** shot noise, Green’s functions, decimation process

## Abstract

We study theoretically the electronic transport and quantum fluctuations in single-molecule systems using thiophene rings as integrated elementary functions, as well as the dependence of these properties with the increase of the coupled rings, i.e., as a quantum wire. In order to analyze the current flow through these molecular systems, the thiophene rings are considered to be connected to metal contacts, which, in general terms, will be related to the application of voltages (bias voltages or gate voltages) to generate non-equilibrium behavior between the contacts. Due to the nonlinear behavior that is generated when said voltages are applied, it is possible to observe quantum fluctuations in the transport properties of these molecular wires. For the calculation of the transport properties, we applied a tight-binding approach using the Landauer–Büttiker formalism and the Fischer–Lee relationship, by means of a semi-analytic Green’s function method within a real-space renormalization (decimation procedure). Our results showed an excellent agreement with results using a tight-binding model with a minimal number of parameters reported so far for these molecular systems.

## 1. Introduction

The great technological revolutions that have been occurred over the years—such as the Industrial Revolution of the nineteenth century and the technological revolution that was based on the first transistor (1947), integrated circuits (1958) [1,2,3], solid state circuits and semiconductors in the twentieth century have had a transformative impact in society, culture, and the scientific community. The scientific and technological transformations that have been caused by the development of integrated circuits have mainly been caused by the miniaturization of their components, based on Moore’s law. Currently, miniaturization is leading to the construction of circuits at the molecular or atomic level, which will open the way to the development of molecular electronics.

Molecular electronics can be defined as a technology that uses individual molecules or small groups of molecules to perform electronic functions [2]. In the 1970s, Aviram reported the preparation and characterization of donor–acceptor (DA) species, his work being one of the pioneers in the field of molecular electronics [4]. This led to the study of load transfer, analyzing energy levels, Highest Occupied Molecular Orbital (HOMO) and Lowest Unoccupied Molecular Orbital (LUMO), where the charge transfer required a threshold voltage. It also led to the development of different types of low molecular dimensional electronic devices [1,5,6,7].

However, new assembly conditions arise, such as junctions formed with small molecules, strongly bonded to two electrodes. These conditions must be carefully considered because the mechanisms or connection devices (wires) must have certain conduction characteristics. This is the reason why much of the previous research has concentrated on the conductance characteristic of conjugated linear oligomers, taking into account properties as: (a) room temperature within a scheme of tunneling transport through the discrete electronic states of the molecule; (b) I−V and conductance characteristics using the Mechanically Controllable Break Junctions (MCBJ), where the molecular system with end-groups reactive is placed inside the contacts so that the electrodes can be adjusted to certain distances comparable to the length of the molecule; (c) point contact method with scanning tunneling microscopy (STM), which enables selectively performing a repeated analysis of the conductance through a specific molecule; (d) analysis of vibrational spectra and photochemical stability of terthiophene based molecular wires through surface-enhanced Raman scattering (SERS); and (e) properties of adsorption, among others [5,8,9,10,11,12,13,14,15,16,17].

In this work, the properties of quantum transport and current fluctuations are analyzed through molecular systems, specifically those systems that have in their molecular structure thiophene rings, which are known as molecular wires i.e., can be considered as solid-state structures of organic charge transfer (CT) with special electronic characteristics ranging from insulators to conductors [7,8,9,10,11,12]. The transport properties are discussed via a theoretical and analytical method based on a renormalization procedure of a molecular system with *N*-thiophene rings taking into account the interactions between the molecule, rings, and contacts. In particular, the study includes molecular wires with one, two, and three thiophene rings placed between two metal contacts. Special attention is devoted to the electron transport by placing the system between metal leads and controlling the nonlinear bias voltage dependence of the current. Associated with such nonlinear character, quantum fluctuations in the transport properties—known as the noise power spectrum—can be observed. The noise power provides relevant information of the electronic correlation via the Fano factor (F), which indicates whether the magnitude of the noise reaches a Poisson (F=1) or sub-Poisson (F<1) limits [18,19,20]. Within a real space renormalization framework, the model calculation uses a tight-binding Hamiltonian in the nearest-neighbor approximation and described by Green’s functions formalism, [19,21,22,23,24]. The study involves transmission, current, shot noise, and Fano factor.

The manuscript is organized as follows: in Section 2, the models based in a tight-binding Hamiltonian for the molecular systems with thiophene rings are introduced. In Section 3, the methodology is described. In Section 4, the results are displayed and analyzed. Finally, the conclusions are given in Section 5.

## 2. Model

**Figure 1:**
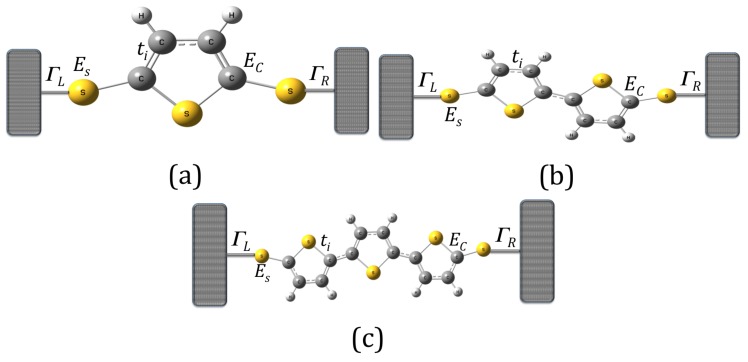
(color online) Molecular models with thiophene rings: (**a**) with one ring (model 1); (**b**) with two rings (model 2); and (**c**) with three rings (model 3).

In order to describe the single-molecule systems using thiophene rings as electronic devices, we have adopted the interaction of each system connected to the left and right contacts, as can bee seen in Figure 1.

The full system is described by a tight-binding Hamiltonian, which is given by:

(1) $$h=h_{Tr}+h_{C}+h_{I},$$

where $h_{Tr}$ corresponds to the Hamiltonian of the molecular system with thiophene rings and embedded between two electrodes and is given by:

(2) $$h_{Tr}=\sum_{i}E_{i}r_{i}^{†}r_{i}+\sum_{i}t_{i}\left(r_{i}^{†}r_{\left(i+1\right)}+r_{\left(i+1\right)}^{†}r_{i}\right)\;,$$

where $r_{i}^{†}$ is the creation operator of an electron at *i* atomic site of the thiophene ring and $E_{i}$ is the energy site of the carbon or sulfur atoms ($E_{c},\;E_{s}$). The parameter $t_{i}$ is the hopping between the *i* atoms, where $t_{\tau }$ is the [C−S] coupling out of the ring, $t_{s}$ is the [C−S] coupling into the ring, $t_{d}$ is the [C=C] coupling, $t_{c}$ is [C−C] coupling into the ring, and $t_{\delta }$ is the [C−C] coupling out of the ring.

The Hamiltonian $h_{C}$ represents the metal contact and $h_{I}$ represents their interaction with the molecular system, given by:

(3) $$h_{C}=\sum_{k_{L}}\xi_{k_{L}}c_{k_{L}}^{†}c_{k_{L}}+\sum_{k_{R}}\xi_{k_{R}}c_{k_{R}}^{†}c_{k_{R}}$$

and

(4) $$h_{I}=\sum_{k_{L}}\Gamma_{L}c_{k_{L}}^{†}r_{L}+\sum_{k_{R}}\Gamma_{R}c_{k_{R}}^{†}r_{R}+h.c.,$$

where the operator $c_{k_{L,R}}^{†}$ is the creation operator of an electron in a state $k_{L,R}$ with energy $\xi_{k_{L,R}}$, while $\Gamma_{L,R}$ is the coupling between each contact (left and right) with the *i* atomic site on the left and right sides of the molecular system.

As we can observe, Equations (2)–(4) represent a many-body problem, where one electron passes through the molecular system interacting electronically with the states of atoms in the molecule or the contacts. Therefore, we do an approximation, where the electron–electron interaction is not taken into account so that this problem of many-body can be considered a scattering problem of a single-electron in a multichannel system.

In this new approach, the molecular states of the system (Left lead–thiophene molecule–Right lead) are expanded into a linear combination of atomic orbitals (LCAO) (*p* orbitals or π-electron for our molecular system). These states are defined by $\phi_{l}=\sum_{m}c_{l}\psi_{m}$, where l,m represent the electronic site in the leads or in the effective molecular system.

## 3. Method

We study the current’s fluctuations through molecular wires composed of thiophene rings connected to two semi-infinite contacts by using the Landauer–Büttiker formalism [18,19,20]. The calculations are based on Green’s function techniques within a real-space renormalization approach for molecular systems with one, two, and three rings of thiophene (Figure 1a–c, respectively). This allows us to reduce the many-body problem into a multichannel scattering problem for a single electron. The Green’s function of the molecular systems coupled to the contacts is related to the tight-binding Hamiltonian *h* through the expression G=1/(E−iη−h), where η is a infinitesimal term; therefore, G can be calculated by using the Dyson equation, given by

(5) $$G=G_{0}+G_{0}\left(\Sigma_{L}+\Sigma_{R}\right)G,$$

where $G_{0}$ is the bare Green’s function of the isolated molecular system and $\Sigma_{L}$ and $\Sigma_{R}$ are the self-energies of the left and right contact, respectively.

The transmission probability can be obtained by using the Fischer–Lee relationship [18]:

(6) $$T\left(E\right)=Tr\left[\Gamma_{L}G^{r}\Gamma_{R}G^{a}\right]\;,$$

where $\Gamma_{L(R)}=i\left(\Sigma_{L(R)}-\Sigma_{L(R)}^{†}\right)$ is the spectral matrix density of the left (right) contact. Here, we use $\Sigma_{L}=\Sigma_{R}=-i\Gamma /2$.

**Figure 2:**
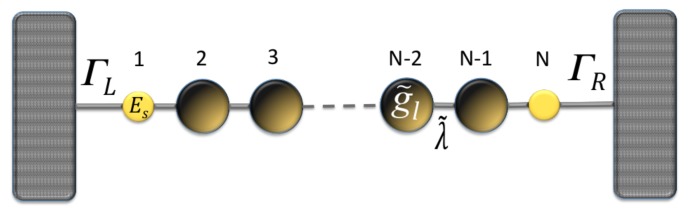
(color online) Representation of the effective molecule coupled with the contacts.

In order to obtain the Green’s functions $G_{0}$, we transform the molecular system with one, two, and three thiophene rings without contacts into an effective one-dimensional chain. This allows us to obtain new renormalized Green’s functions that contain all the information of the planar molecule. The transmission probability for the new effective molecular one-dimensional system (as shown Figure 2) can then be written as:

(7) $$T\left(E\right)=4\Gamma_{L}\Gamma_{R}{\left|G_{1N}\right|}^{2},$$

with

(8) $$G_{1N}=\frac{G_{1N}^{0}}{\left(1-\Sigma_{L}G_{11}^{0}\right)\left(1-\Sigma_{R}G_{NN}^{0}\right)-\Sigma_{L}\Sigma_{R}{\left(G_{1N}^{0}\right)}^{2}},$$

where $G_{1N}$ has been calculated from Equation (5) (including the contacts) given by: $G_{1N}=G_{1N}^{0}+G_{11}^{0}\Sigma_{L}G_{1N}+G_{1N}^{0}\Sigma_{R}G_{NN}$.

On the other hand, the Green’s function $G_{1N}^{0}$, $G_{NN}^{0}$, and $G_{11}^{0}$ can be analytically determined by using renormalization techniques (the details of the Green’s function calculations are presented in Appendix A). With $G_{NN}^{0}=G_{11}^{0}$ and $\Gamma_{L}=\Gamma_{R}=\Gamma$, we can rewrite Equation (7) as:

(9) $$T\left(E\right)=\frac{4\Gamma^{2}{\left(G_{1N}^{0}\right)}^{2}}{{\left|{\left[1+\frac{i\Gamma }{2}G_{NN}^{0}\right]}^{2}+\frac{\Gamma^{2}}{4}{\left(G_{1N}^{0}\right)}^{2}\right|}^{2}}\;.$$

Once we have calculated the transmission probability, we calculate the conductance given by:

(10) $$G=G_{0}T\left(E\right),$$

where $G_{0}$ is defined as the quantum of conductance and is given by $G_{0}=2e^{2}/h$. In the coherent transport regime, where the location length is larger than the length of the conductor system, we determine the current that passes through the molecular systems, which is considered as a scattering process of an electron between the contacts. Using the Landauer’s formalism, the I−V characteristics can be obtained by the expression [18,19]:

(11) $$I\left(V\right)=I_{o}\int_{-\infty }^{\infty }\left(f_{L}-f_{R}\right)T\left(E\right)dE\;,$$

where $I_{o}=e/\pi \hbar$ and $f_{L(R)}$ is the Fermi–Dirac distribution function given by $f_{L(R)}=f\left(E-\mu_{L(R)}\right)$, where $\mu_{L(R)}=E_{f}\pm eV/2$ is the chemical potential.

The noise power of current fluctuations (NPCF) is calculated by the expression [20,25,26]

(12) $$S=S_{o}\int_{-\infty }^{\infty }T\left(E\right)\left[f_{L}\left(1-f_{L}\right)+f_{R}\left(1-f_{R}\right)+\left\{\left(1-T\left(E\right)\right){\left(f_{L}-f_{R}\right)}^{2}\right\}\right]dE\;,$$

where $S_{o}=2e^{2}/\left(\pi \hbar \right)$. The equilibrium noise contribution is given by the first two terms of this Equation (12), and the last term gives the non-equilibrium or shot noise contribution to the power spectrum. We can determinate the Fano factor *F* by calculating the NPCF (*S*) and the total current flowing through the aromatic molecules, with the following relationship [20]:

(13) $$F=\frac{S}{2eI}\;.$$

For F=1, the shot noise achieves the Poisson limit for which there is no correlation between the charge carriers. On the other hand, for F<1, the shot noise achieves the sub-Poisson limit and it provides the information about the quantum correlation of the charge carriers [27,28].

The next section describes the analytical and numerical results obtained from the application of the decimation method and Landauer’s formalism for molecular systems with one, two, and three thiophene rings connected to terminal electrodes.

## 4. Results

The calculation of the transmission probability T(E) of the electron through the molecular system is essential if we wish to calculate the other transport properties such as conductance, current, and current fluctuations, where we have considered variations in the coupling between the contacts $\Gamma_{L(R)}$ and the molecular system.

**Figure 3:**
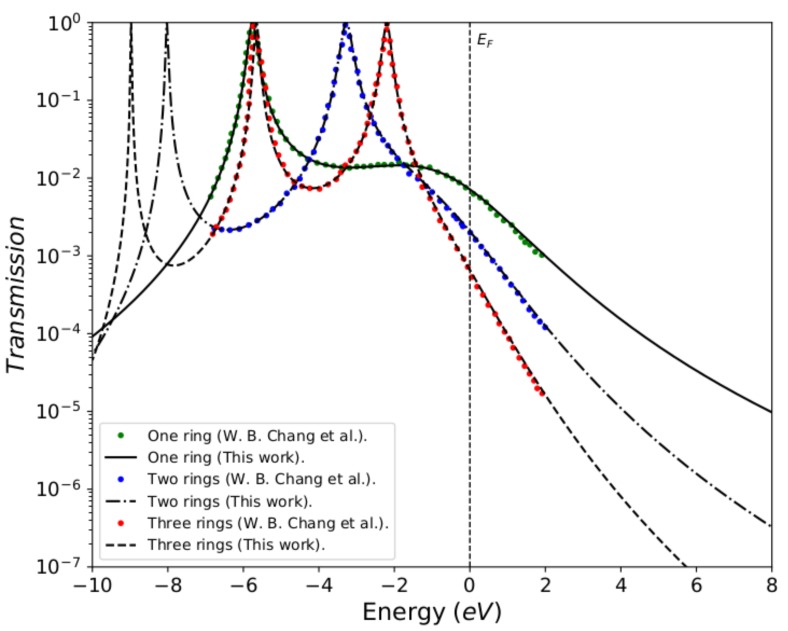
Transmission probability T(E) as a function of the injection energy of the electron *E*, for the molecular systems with one, two, and three thiophene rings for the values: Γ=5.32 eV, $E_{b}=-5.54$ eV, $E_{s}=-0.98$ eV, δ=−2.40 eV and τ=−0.83 eV.

To validate the method used in this work, the reproduction of the reported calculations for the molecular systems with thiophene rings determined by a tight-binding numerical model with a minimal number of parameters. Figure 3 shows the transmission probability T(E) as function of the injection energy of the electron *E*, which compares the calculations made in this work with the results reported by Chang et al. [5]. The black lines represent the transmission probability calculated by Green’s functions (analytical method), and the green, blue, and red lines represent the transmission probability calculated by Green’s functions based on the numerical model for the systems composed of one, two, and three thiophene rings, respectively. We can observe that the calculations performed here (black lines) perfectly fit the reported calculations, which confirm the validity of using Green’s functions in this work.

The profile in the transmission probability shows *N* resonant peaks, which represent the eigenvalues for each molecular system, where each ring is modeled as an atomic site (or quantum dot) with an energy site of $E_{b}$.

We have taken the same energy values from Chang et al., where $E_{b}=-5.54$ eV (thiophene ring energy), $E_{S}=-0.98$ eV (sulfur atom energy), τ=−0.83 eV (sulfur-ring coupling), δ=−2.40 eV (rings coupling), and Γ=−5.32 eV (molecule–electrode coupling) [5]. However, in our calculations, we have taken the largest energy width so that we can observe the *N* resonances, which must correspond to the molecular systems with *N* thiophene rings.

If we have a system with a thiophene ring, then there will be a resonant peak corresponding to the eigenvalue of its own state, which is at approximately −5.54 eV. When we have two rings, the bandwidth in the transmission probability is extended. This results in two resonances that coincide with the eigenvalues of the states of each of the thiophene rings, being −3.4 eV and −7.6 eV approximately. Finally, when the molecular system contains three rings, the profile in the transmission probability presents three resonant peaks that correspond to the eigenvalues of the three rings, being found in −2.0 eV, −5.54 eV and −9 eV approximately, as also, they are calculated by diagonalizing the Hamiltonian of the molecular system (Equation (2)).

The behavior of the profile in the transmission probability shows that as the number of thiophene rings increases, the width of the band also increases. This allows the position of the eigenstates of the system, particularly the state associated with the Highest Occupied Molecular Orbital (HOMO), to get close to the Fermi level. However, the amplitude of the transmission probability decreases as the number of rings increases. This behavior physically represents a decrease in the overlap between the charge density of the border states (sulfur states) and the thiophene rings.

**Figure 4:**
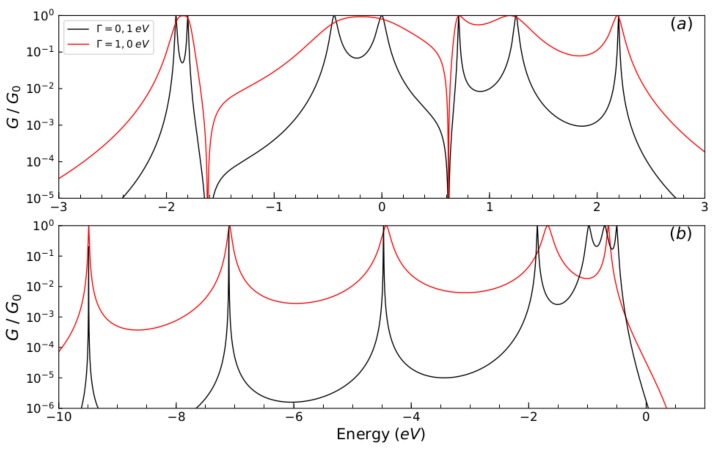
(color online) Normalized conductance ($G/G_{0}$) as function of the injection energy of the electron *E*, for the molecular system with one thiophene ring for different values of Γ: (**a**) $E_{c}=E_{s}=0$ eV, $t_{\tau }=t_{d}=t_{c}=t_{\delta }=t_{s}=1.0$ eV, (Black line) Γ=0.1 eV and (red line) Γ=1.0 eV and (**b**) $E_{c}=-5.54$ eV, $E_{s}=0.98$ eV, $t_{d}=t_{c}=t_{\delta }=-2.40$ eV, $t_{\tau }=t_{s}=-0.83$ eV, (Black line) Γ=0.1 eV and (red line) Γ=5.32 eV.

About the comparison between the present results and those from Chang et al., we have to stress that both models should arrive essentially to the same results. The theoretical model presented in [5] corresponds to the limit of our model when each thiophene ring is viewed as one site. In effect, the parameters that were taken in our model were adjusted taking into account the work of Chang et al. This is due to the fact that, as it was aimed for, when comparing the two methods, the results were perfectly adjusted (as it was observed in Figure 3). Once the accuracy of the results had been verified, we took another set of parameters, not only to verify the model, but also for the behavior in the conduction bands, taking into account the variation of the molecule-contact coupling (Γ). The hybridization behavior was checked by widening the levels in a strong coupling regime, resulting in an increase in the amplitude in the current intensity. On the other hand, the model presented in this paper verifies in more detail the molecular electronic structure, which was also done by calculating the eigenvalues of the system, resulting in the fact that the number of resonances presented in the conductance profile (Figure 4) matches the number of carbon atoms in each thiophene ring.

When our method has been validated, we then analyze the conductance, current, and shot noise through molecular wires composed of thiophene rings, considering the variation of the length of the molecular system and also the coupling between the contacts ($\Gamma_{L,R}=\Gamma$) and the molecular system.

First, we study the behavior of the normalized conductance as a function of energy having accounted for two sets of values in energy site of each carbon and sulfur atoms and their corresponding couplings, given by: $E_{c}=E_{s}=0$ eV, $t_{\tau }=t_{d}=t_{c}=t_{\delta }=t_{s}=1.0$ eV for the first set of values; and $E_{c}=-5.54$ eV, $E_{s}=0.98$ eV, $t_{d}=t_{c}=t_{\delta }=-2.40$ eV, $t_{\tau }=t_{s}=-0.83$ eV for the second set of values. We have taken the set of molecular parameters (first set of values) to validate our real space renormalization model. We remark that these sets of molecular parameters (second set of values) are in the range of the parameters that are used to obtain the results calculated by Chang et al. using the numerical model of tight-binding. They are also consistent with the experimental results [5].

The results of normalized conductance ($G/G_{0}$) as a function of energy for a molecular wire with one thiophene ring for different values of energy of carbon atoms ($E_{c}$), sulfur atoms ($E_{s}$), carbon–carbon coupling ($t_{d},\;t_{c},\;t_{\delta }$), sulfur–carbon coupling ($t_{\tau },\;t_{s}$), and different Γ coupling values are displayed in Figure 4.

We observe that conductance is a regime of weak coupling ($t_{\tau },\;t_{\delta },\;t_{s},\;t_{c},\;t_{d},\;⩾\Gamma$) and the molecular system is a resonant tunneling regime; on the other hand, we find a regime of strong coupling ($t_{\tau },\;t_{\delta },\;t_{s},\;t_{c},\;t_{d},\;\leq \Gamma$), where the width of the peaks in the transmission have increased due to the hybridization of the energy levels of the end sites of the molecule and the continuum of the lead states. The effect of the contacts on the quantum system is one of the main features that regulates the transport properties [29,30,31]. Therefore, *N* unitary normalized conductance peaks are displayed (Figure 4a,b, black lines), which can be associated with the eigenvalues of system’s Hamiltonian in a matrix form of Equation (2) given by:

(14) $$h_{Tr}=\left(\begin{array}{ccccccc} E_{s} & t_{\tau } & 0 & 0 & 0 & 0 & 0\\ t_{\tau } & E_{c} & t_{s} & 0 & 0 & t_{d} & 0\\ 0 & t_{s} & E_{s} & t_{s} & 0 & 0 & 0\\ 0 & 0 & t_{s} & E_{c} & t_{d} & 0 & t_{\tau }\\ 0 & 0 & 0 & t_{d} & E_{c} & t_{c} & 0\\ 0 & t_{d} & 0 & 0 & t_{c} & E_{c} & 0\\ 0 & 0 & 0 & t_{\tau } & 0 & 0 & E_{s} \end{array}\right).$$

The eigenvalues that are shown in Figure 4a are given by 2.19 eV, −1.99 eV, −1.8 eV, 1.25 eV, 0.71 eV, −0.45 eV, and 0 for the first set values and in Figure 4b are given by −9.49 eV, −7.11 eV, −4.47 eV, −1.85 eV, −0.98 eV, −0.70 eV, and −0.49 eV for the second set of values, respectively.

**Figure 5:**
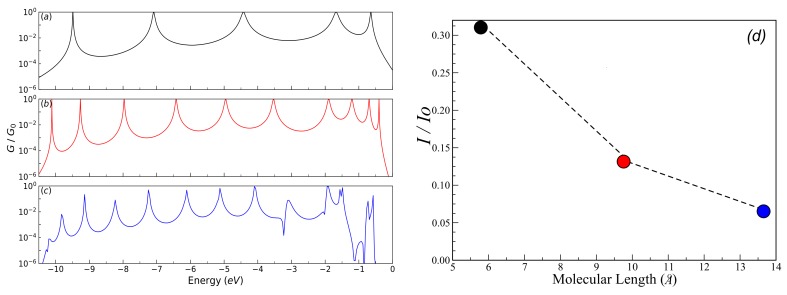
(color online) Normalized conductance ($G/G_{0}$) for (**a**) one ring; (**b**) two rings; and (**c**) three rings as function of energy; (**d**) presents the current (I) as a function of molecular length. Calculations are for $E_{c}=-5.54$ eV, $E_{s}=0.98$ eV, $t_{d}=t_{\delta }=t_{c}=-2.40$ eV, $t_{\tau }=t_{s}=-0.83$ eV, and Γ=5.32 eV.

Figure 5 shows the normalized conductance ($G/G_{0}$) for (a) one ring; (b) two rings; and (c) three rings; (d) depicts the current ($I/I_{o}$) as function of the molecular length. The results are for $E_{c}=-5.54$ eV, $E_{s}=0.98$ eV, $t_{d}=t_{\delta }=t_{c}=-2.40$ eV, $t_{\tau }=t_{s}=-0.83$ eV, and Γ=5.32 eV. We can observe that the number of resonances in the normalized conductance profile increases as the number of rings in the molecular system increases (see Figure 5a–c respectively). However, the normalized conductance amplitude decreases as the number of rings increases (i.e., $G/G_{0}<1$). This behavior can be verified at the maximum amplitude for the current as a function of length (Figure 5d), where the current also decreases with the length and the system will behave as an insulating system, and there will be no charge conduction through the molecular wire, agreeing with the behavior with the conductance depending on the length of the molecular wire determined in the measurements of Capozzi et al. [32].

The molecular distances used in this work were obtained from anionic structures, (-2) S−(thiophene)n−S with N=1,2 and 3, optimized in the Gaussian09 program (Rev. C.01) [33], using the density functional theory (DFT) method B3lyp/6-311+G (2d,p). We performed these geometry optimizations in our scalable computational cluster named Atomic (a team of multiprocessors in support of chemical calculations) using standard gradient techniques by simultaneous relaxation of all geometric parameters. No imaginary frequencies were obtained for these structures, thus confirming their identity as minimums in the potential energy surface.

**Figure 6:**
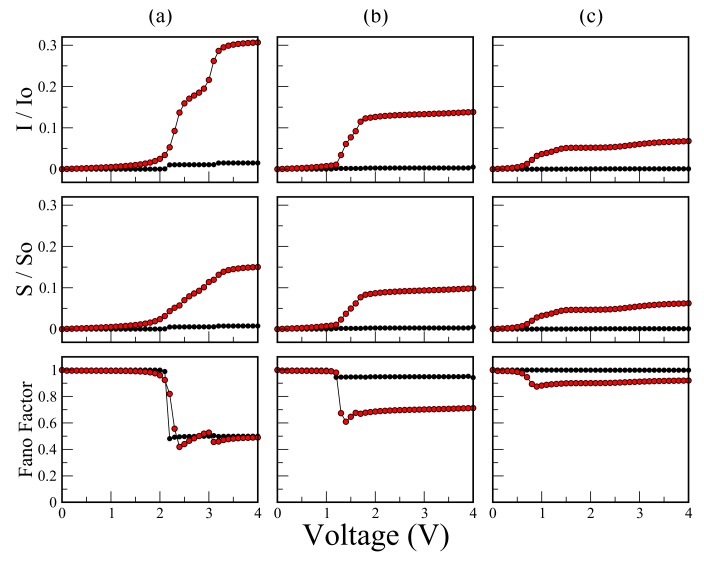
(color online) I−V characteristic ($I/I_{o}$), shot noise ($S/S_{o}$), and Fano factor as a function of bias voltage for the molecular system with (**a**) one thiophene ring; (**b**) two thiophene rings; and (**c**) three thiophene rings. Calculations are for $E_{c}=-5.54$ eV, $E_{s}=0.98$ eV, $t_{d}=t_{\delta }=t_{c}=-2.40$ eV, $t_{\tau }=t_{s}=-0.83$ eV values and (Black dots) Γ=0.1 eV, and (red dots) Γ=5.32 eV.

Figure 6 shows the I−V characteristic ($I/I_{o}$), shot noise ($S/S_{o}$), and Fano factor as a function of bias voltage for the molecular system with (a) one thiophene ring; (b) two thiophene rings; and (c) three thiophene rings for $E_{c}=-5.54$ eV, $E_{s}=0.98$ eV, $t_{d}=t_{\delta }=t_{c}=-2.40$ eV, $t_{\tau }=t_{s}=-0.83$ eV values and (Black dots) Γ=0.1 eV and (red dots) Γ=5.32 eV.

We find two limits of strong (red dots) and weak (black dots) couplings. In the limit of strong coupling, the amplitude of the current decreases as the number of thiophene rings in the molecular system increases. If we take the couplings between atoms of molecule ∼(0.1–6.0) eV, then the maximum amplitude of the current is on the order of microamperes (μA). This is consistent for typical values taken from experiments of maximal current for molecular systems [34]. Another important aspect is that the amplitudes of the current ($I/I_{o}$) and shot noise ($S/S_{o}$) in the strong coupling regime (red dots in Figure 6) increase significantly when compared with the case of weak coupling (black dots in Figure 6), which can be clearly visible by observing the area under the curve of the conductance spectrum at those limits. In the curves of Fano factor (*F*), the shot noise goes from the Poisson limit (F=1) up to the sub-Poisson limit (F<1), as long as the threshold voltage that is determined by the current gap is exceeded. This behavior reveals that the electrons are correlated after the tunneling process has occurred. Note that here the electrons are correlated only in the sense that one electron feels the existence of the other electron according to the Pauli exclusion principle (since all other electron–electron interactions in the formalism have been disregarded). Likewise, after the threshold voltage is exceeded, a greater decrease in the Fano factor is observed for the strong coupling compared with the behavior for the weak coupling, which shows that there is a greater electronic correlation in the strong regime. It is notable that, for the weak coupling regime (Γ=0.1eV), although the threshold voltage is exceeded, the electronic correlation is quite low (F∼1) and decreases with the increase of coupled rings. This is reflected with the amplitudes in the quantum noise and in the current.

**Figure 7:**
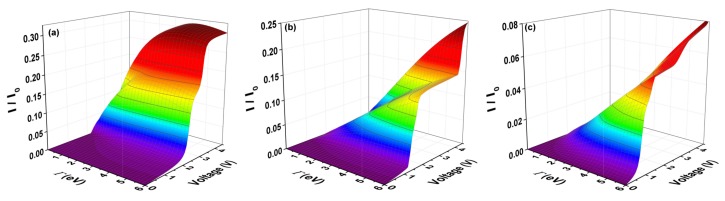
(color online) I−V characteristic $I/I_{o}$ as a function of bias voltage and the molecule–electrode coupling (Γ) for the molecular system with (**a**) one ring; (**b**) two rings; and (**c**) three rings for $E_{c}$ = −5.54 eV, $E_{s}=0.98$ eV, $t_{d}=t_{\delta }=t_{c}=-2.40$ eV, and $t_{\tau }=t_{s}=-0.83$ eV.

In Figure 7, the current is calculated for molecular systems with one thiophene ring (Figure 7a), two thiophene rings (Figure 7b), and three thiophene rings (Figure 7c) through the expression (11). The normalized current ($I/I_{o}$) is shown with $I_{o}=2\;e/h$, as a function of voltage and molecule–electrode coupling (Γ). Figure 7 shows that there is a voltage gap before there is current flow in these systems. This gap indicates if the molecular system is an insulator, semiconductor or conductor (from where the system will begin to flow current with a very low input voltage) and can be observed in the purple zone.

The behavior of the current versus voltage curves indicates that, as the number of rings increases, the amplitude of the threshold current decreases. However, when the molecule–electrode coupling increases (no matter if the amplitude of the current decreases), the gap of voltage decreases with the number of rings or length of the system. This means that, as the molecule–electrode coupling increases, the molecular system can pass from semiconductor (as seen for all systems with one, two and three rings for a weak coupling) to a conductor system for a strong coupling, despite the fact that the amplitude of the threshold in the current decreases. The maximum amplitude of the current (saturation or threshold) of the systems happens for a voltage around 4.5 V and this amplitude then decreases with the increase of the thiophene rings, being 0.30 μA, 0.25 μA, and 0.08 μA for the molecular system with one, two and three rings, respectively.

**Figure 8:**
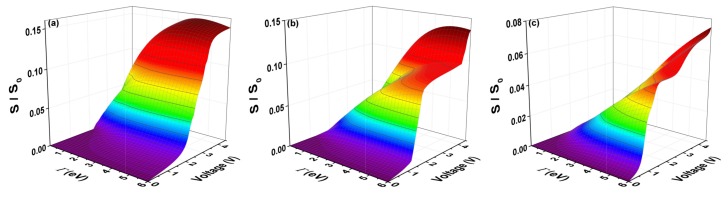
(color online) Shot noise $\left(S/S_{o}\right)$ as a function of bias voltage and the molecule–electrode coupling (Γ) for the molecular system with thiophene rings with (**a**) one ring; (**b**) two rings; and (**c**) three rings for $E_{c}=-5.54$ eV, $E_{s}=0.98$ eV, $t_{d}=t_{\delta }=t_{c}=-2.40$ eV, and $t_{\tau }=t_{s}=-0.83$ eV.

The quantum current fluctuations (*S*) are calculated using the expression (12). Figure 8 shows the normalized current fluctuations ($S/S_{o}$) with $S_{o}=2\;e^{2}/h$, as a function of the bias voltage, for which we have taken a window of the electrode-coupling energy values (Γ) as was done for current curves ($I/I_{o}$). Figure 8 shows that there are certain maximum values of the quantum current fluctuations (*S*) that occur for an average value of the transmission probability and correspond to Γ>> compared to the couplings inter-sites of the molecule and at temperature θ∼0 within the width of the band in the transmission probability that matches when amplitude equals T(E)=1/2. The strong or weak coupling mentioned above also affects the amplitude of the shot noise, which decreases when the coupling between molecule and contacts is very small.

**Figure 9:**
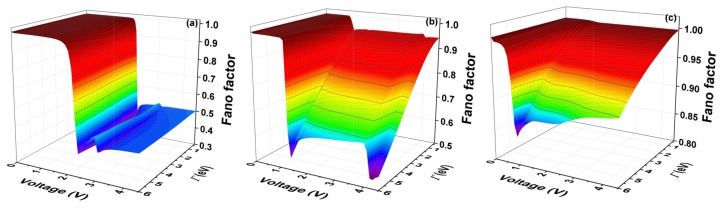
(color online) Fano factor (F) as a function of bias voltage and the molecule–electrode coupling (Γ) for the molecular system with thiophene rings with (**a**) one ring (**b**); two rings (**c**); and three rings for $E_{c}=-5.54$ eV, $E_{s}=0.98$ eV, $t_{d}=t_{\delta }=t_{c}=-2.40$ eV, and $t_{\tau }=t_{s}=-0.83$ eV.

The Fano factor (*F*) is calculated using the expression (13). Figure 9 shows the Fano factor as a function of the voltage and the molecule–electrode coupling (Γ), for the systems with one ring, two rings, and three rings, respectively.

The Fano factor indicates the correlation between the electrons of the system. Therefore, Figure 9 shows that, for small voltages, the electronic correlation of the system is zero (F=1). However, the correlation grows proportionally with the increase of the coupling Γ, (F<1). We see, therefore, that the quantum noise goes from a Poissonian limit (F=1) to a sub-Poissonian limit (F<1) as it increases both Γ and the voltage. When (F<1), the electrons are correlated and, therefore, a tunneling process will be observed.

## 5. Conclusions

The transport properties and current fluctuations were determined through molecular wires composed of one, two, and three rings of thiophene using the decimation method and the Green’s functions technique within the tight-binding model. This method was verified with the conductance profile in which the eigenvalues of the system coincided with the resonance peaks. Is important to note that, although in this work a comparison of our analytical results with the numerical results given by Chang et. al. was made, this method has been used to compare transport properties through molecular systems determined by means of Density Functional Theory (DFT) method and/or experimental results [22,24,35].

The renormalization method, which transforms the real space to an effective space, is suitable to study the transport properties in molecular systems, especially those that are large or have high complexity level of symmetry. As we could see, the idea of reducing a set of linear equations to nonlinear equations with energy and effective couplings (resulting a Schrödinger equation of the form $\tilde{E}\tilde{\psi }-{\tilde{h}}_{Tr}\tilde{\psi }=0$), can generate an elimination of variables, degrees of freedom and therefore the systematic reduction of the dimension of Hamiltonian; however, this process involves a high calculation cost.

For the reason above, we follow the perturbation theory where the renormalization process of the system is equivalent to the inclusion of Feynman’s paths, using Green’s functions, which can be considered as propagators carrying information about the quantum transport properties of the molecular system. These functions provide an alternative way to analyze the solutions of the Schrödinger equation, providing numerical and analytically stable solutions; in contrast to the solutions that contain a Hamiltonian $h_{Tr}$ of order *N*, where *N* is the number of atoms in the molecular system, which grows as the number of rings increases and therefore can generate numerically unstable solutions.

In this direction, the transmission probability, the characteristic curves I−V ($I/I_{0}$), shot noise, and Fano factor for different coupling values between the molecular systems with the electrodes (Γ), were calculated under two specific regimes: (i) a weak coupling regime (Γ=0.1 eV), which corresponds to resonant tunnelling where the resonances are associated to the electronic state energies confined in a molecular wire of N atomic sites and (ii) a strong coupling regime (Γ=5.32 eV), where the electronic structure is significantly modified and leading to displaced and amplified resonances that are not related to the isolated molecular levels, but with the hybridization between the final sites of the chain and the contacts. In the last regime, the transmission is at a maximum for all energies within the side bands. Therefore, the great influence of the Γ coupling on the transport properties, of the molecular systems, induced higher current amplitudes and greater electronic correlation for each system. This correlation refers to the fact that the state of the electron moving through the molecular system is not independent of the electronic states, and therefore a tunneling process can be generated. In addition, the current amplitude (I) declined with the increase in the number of rings coupled to the system. This indicates that there was a decline in the electronic correlation (and an increase in the Fano factor).

As the number of rings increased, the systems presented a decay in the amplitude of current and also a small decrease in the threshold voltage.

On the other hand, in the strong coupling regime, quantum transport is dominated by a tunneling called “Landauer transport regime”. This regime is also known as coherent tunneling and is characterized by the alignment (hybridization) of the orbitals of the atoms at the end of the molecule with the Fermi level of leads. This behavior can be of interest because these kinds of sulphur systems (i.e., with sulfur atoms at the ends of the molecular system) can be taken into account as possible prototypes for devices that can be used in molecular electronics, due to the strong coupling between S-lead, which causes a large location of charge in the interface. These molecular systems can be taken with S−Au junctions, for example, where the S-lead coupling can be between 1 eV and 4 eV [36,37], values that for our molecular systems are taken as strong couplings.

The above confirm the effectiveness of our method and thus gives us the feasibility of implementing it in future work to analyze transport properties in other molecular devices, or to calculate other thermal properties depending on wire length, properties of spin filter, among others, through molecular wire composed of thiophene rings.

## Figures and Tables

**Figure A1:**
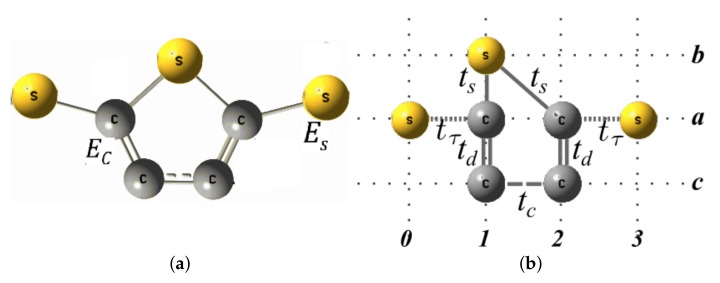
Molecular system (**a**) and geometric model (**b**) with a thiophene ring without electrodes.

**Figure A2:**
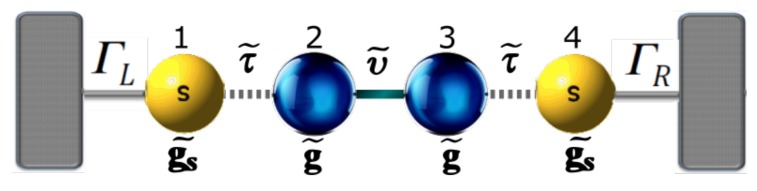
Molecular system with a renormalized thiophene ring.

**Figure A3:**
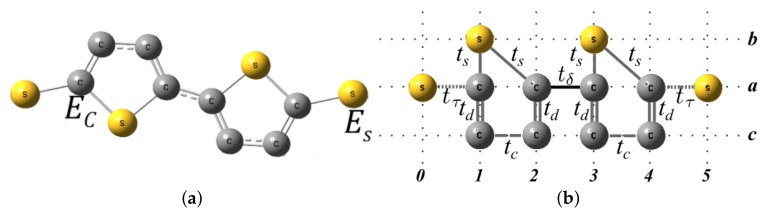
Molecular system (**a**) and geometric model (**b**) with two Thiphene rings without electrodes.

**Figure A4:**
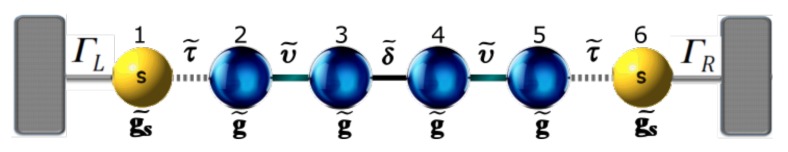
Molecular system with two renormalized thiophene rings.

**Figure A5:**
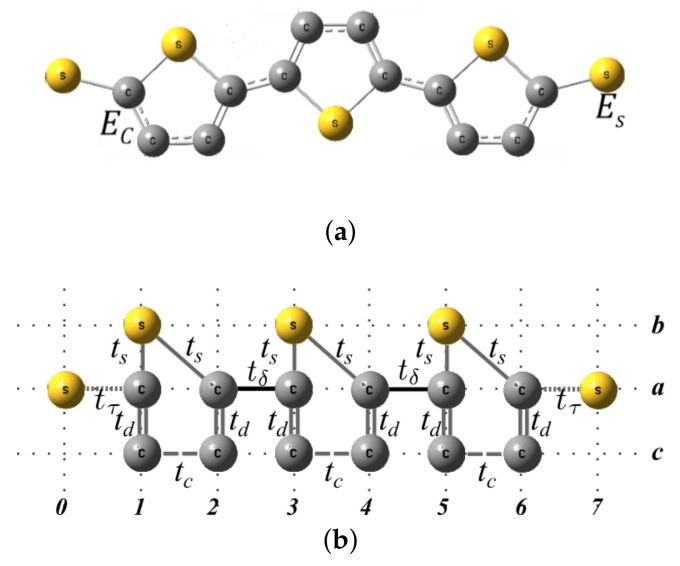
Molecular system (**a**) and geometric model (**b**) with three Thiphene rings without electrodes.

**Figure A6:**
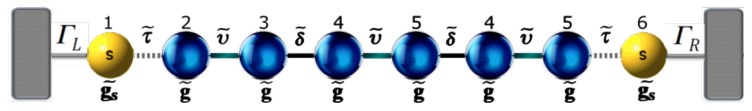
Molecular system with three renormalized thiophene rings.

## Appendix A. Renormalization Process and Green’s Function for Molecular Systems with One, Two and Three Thiophene Rings

### Appendix A.1. Renormalization Process for Molecular Systems with One Thiophene Ring

The molecular system contains carbon and sulfur atoms with energy per site $E_{C}$ and $E_{S}$, respectively (see Figure A1a). These can be reorganized geometrically as shown in Figure A1b to facilitate the calculation of Green’s functions. As we can see, we have labeled horizontally the sites 0, 1, 2 and 3 that will be renormalized, and we have labeled vertically the linear chains as a, b, and c, which are vertically coupled by the intrasites 0, 1, 2, or 3. The information that has the chains b and c will be will be renormalized towards the chain a. Since the sites 0 and 3 only contain a single atom, that is, they are not vertically coupled with other atoms, these sites are not necessary to renormalize them, therefore, the sites 1 and 2 only are renormalized.

Each atomic site has an associated undisturbed local Green function given by $g_{i}=\frac{1}{E-E_{i}}$, where *i* represents the carbon (*C*) or sulfur (*S*) atoms, so the use of the Dyson (5) equation allows us to write the equations of motion of the electron at each site with the interaction to first neighbors.

To perform the decimation, a first set of equations is determined, where the sites with subscript one (1) are taken into account as follows:

(A1) $$G_{11}^{a}=g_{c}+g_{c}t_{\tau }G_{10}^{a}+g_{c}t_{s}G_{11}^{b}+g_{c}t_{d}G_{11}^{c},$$

(A2) $$G_{11}^{b}=g_{s}t_{s}G_{11}^{a}+g_{s}t_{s}G_{12}^{a},$$

(A3) $$G_{11}^{c}=g_{c}t_{d}G_{11}^{a}+g_{c}t_{c}G_{12}^{c},$$

(A4) $$G_{12}^{c}=g_{c}t_{c}G_{11}^{c}+g_{c}t_{d}G_{12}^{a}.$$

To solve the system of equations, we first substitute Equation (A4) in (A3), obtaining:

(A5) $$G_{11}^{c}=\frac{g_{c}t_{d}}{\left[1-{\left(g_{c}t_{c}\right)}^{2}\right]}G_{11}^{a}+\frac{g_{c}^{2}t_{c}t_{d}}{\left[1-{\left(g_{c}t_{c}\right)}^{2}\right]}G_{12}^{a}.$$

Likewise, by replacing Equations (A2) and (A5) in Equation (A1), we get $G_{11}^{a}$:

(A6) $$G_{11}^{a}=\frac{g_{c}\left[1-{\left(g_{c}t_{c}\right)}^{2}\right]}{\left[1-{\left(g_{c}t_{c}\right)}^{2}-g_{c}g_{s}t_{s}^{2}\left[1-{\left(g_{c}t_{c}\right)}^{2}\right]-{\left(g_{c}t_{d}\right)}^{2}\right]}+\frac{g_{c}t_{\tau }\left[1-{\left(g_{c}t_{c}\right)}^{2}\right]G_{10}^{a}}{\left[1-{\left(g_{c}t_{c}\right)}^{2}-g_{c}g_{s}t_{s}^{2}\left[1-{\left(g_{c}t_{c}\right)}^{2}\right]-{\left(g_{c}t_{d}\right)}^{2}\right]}+\frac{\left[g_{c}g_{s}t_{s}^{2}\left[1-{\left(g_{c}t_{c}\right)}^{2}\right]+g_{c}^{3}t_{c}t_{d}^{2}\right]G_{12}^{a}}{\left[1-{\left(g_{c}t_{c}\right)}^{2}-g_{c}g_{s}t_{s}^{2}\left[1-{\left(g_{c}t_{c}\right)}^{2}\right]-{\left(g_{c}t_{d}\right)}^{2}\right]}.$$

Analyzing the previous relationship, we observe that it has the form of a Dyson equation. In this case, we obtain the Green’s functions for effective site 1, which is coupled with effective site 0 through an effective coupling $\tilde{\tau }$, and with the effective site 2 through an effective coupling ${\tilde{\upsilon }}_{12}$, as follows:

(A7) $$G_{11}^{a}=g_{1}+g_{1}\tilde{\tau }G_{10}^{a}+g_{1}{\tilde{\upsilon }}_{12}G_{12}^{a}.$$

By comparing Equations (A6) and (A7), the following relationships are found:

The first independent term corresponds to the effective local Green’s function of site 1 ($g_{1}$):

(A8) $$g_{1}=\frac{g_{c}\left[1-{\left(g_{c}t_{c}\right)}^{2}\right]}{\left[1-{\left(g_{c}t_{c}\right)}^{2}-g_{c}g_{s}t_{s}^{2}\left[1-{\left(g_{c}t_{c}\right)}^{2}\right]-{\left(g_{c}t_{d}\right)}^{2}\right]}.$$

Now, knowing that $g_{1}$ is given by Equation (A8), and by comparing the second terms of Equations (A6) and (A7), we get the relationship between the coupling $t_{\tau }$ and effective coupling $\tilde{\tau }$.

$$\tilde{\tau }=t_{\tau }.$$

Similarly, the relationship for the effective coupling between sites 1 and 2 is obtained by comparing the third terms of Equations (A7) and (A8):

(A9) $${\tilde{\upsilon }}_{12}=\frac{g_{s}t_{s}^{2}\left[1-{\left(g_{c}t_{c}\right)}^{2}\right]+g_{c}^{2}t_{c}t_{d}^{2}}{1-{\left(g_{c}t_{c}\right)}^{2}}.$$

Then, to perform the decimation of site 2 of the effective chain and in a similar way as was done for site 1, a second system of equations is proposed of the form:

(A10) $$G_{22}^{a}=g_{c}+g_{c}t_{s}G_{21}^{b}+g_{c}t_{d}G_{22}^{c}+g_{c}t_{\tau }G_{23}^{a},$$

(A11) $$G_{21}^{b}=g_{s}t_{s}G_{22}^{a}+g_{s}t_{s}G_{21}^{a},$$

(A12) $$G_{22}^{c}=g_{c}t_{d}G_{22}^{a}+g_{c}t_{c}G_{21}^{c},$$

(A13) $$G_{21}^{c}=g_{c}t_{c}G_{22}^{c}+g_{c}t_{d}G_{21}^{a}.$$

By substituting (A13) in (A12), we get:

(A14) $$G_{22}^{c}=\frac{g_{c}t_{d}}{\left[1-{\left(g_{c}t_{c}\right)}^{2}\right]}G_{22}^{a}+\frac{g_{c}^{2}t_{c}t_{d}^{2}}{\left[1-{\left(g_{c}t_{c}\right)}^{2}\right]}G_{21}^{a}.$$

Also, using the expressions (A14) and (A11) in (A10), we obtain:

(A15) $$G_{22}^{a}=g_{c}+g_{c}t_{\tau }G_{10}^{a}+g_{c}t_{s}\left(g_{s}t_{s}G_{11}^{a}+g_{s}t_{s}G_{12}^{a}\right)+g_{c}t_{d}\left(\frac{g_{c}t_{d}}{\left[1-{\left(g_{c}t_{c}\right)}^{2}\right]}G_{11}^{a}+\frac{g_{c}^{2}t_{c}t_{d}}{\left[1-{\left(g_{c}t_{c}\right)}^{2}\right]}G_{12}^{a}\right).$$

It is evident that the expressions (A6) and (A15) are equal, therefore:

(A16) $$\begin{array}{c} g_{1}=g_{2}=\tilde{g},\;\tilde{\tau }=t_{\tau },\;{\tilde{\upsilon }}_{12}={\tilde{\upsilon }}_{21}=\tilde{\upsilon }. \end{array}$$

With the realization of the decimation procedure, an effective linear system is obtained with the same information as used in the original system, but, in this case, with four sites, such as the one shown in Figure A2, where each effective site is described by the local Green’s functions $\tilde{g}$ with effective links $\tilde{\tau }$ and $\tilde{\upsilon }$.

Taking into account that the resulting effective system (see Figure A2), is equivalent to the real, linear and symmetric system, the expression (9) can be used to determine the transmission function. However, for its application, it is necessary to find the functions $G_{11}^{0}$, $G_{1N}^{0}$ and $G_{NN}^{0}$ of the system, ($G_{11}^{0}$ , $G_{1N}^{0}$ and $G_{NN}^{0}$ are Green’s functions that are not perturbed by the electrodes, but which present disturbances to their first neighbors), which are determined by the Dyson equation; therefore, we can rewrite Equation (5) as:

(A17) $${\tilde{g_{l}}}^{-1}G_{lm}^{0}=\delta_{lm}+{\tilde{\lambda }}_{m,m-1}G_{l,m-1}^{0}+{\tilde{\lambda }}_{m,m+1}G_{l,m+1}^{0},$$

where l,m represent the sites of the lineal effective chain, $\tilde{g_{l}}$ is the effective local green function and $\tilde{\lambda }$ is the effective coupling between first neighbors of the effective chain.

As shown in Figure A2 and using Equation (A17) the result are four sites; hence, we have to calculate $G_{11}^{0}$, $G_{1N}^{0}$ and $G_{NN}^{0}$ with N=4, obtaining:

$$\begin{array}{cc} G_{14}^{0} & =\tilde{g_{s}}\tilde{\tau }G_{24}^{0},\\ G_{24}^{0} & =\tilde{g}\tilde{\upsilon }G_{34}^{0}+\tilde{g}\tilde{\tau }G_{14}^{0},\\ G_{34}^{0} & =\tilde{g}\tilde{\tau }G_{44}^{0}+\tilde{g}\tilde{\upsilon }G_{24}^{0},\\ G_{44}^{0} & =\tilde{g_{s}}+\tilde{g_{s}}\tilde{\tau }G_{34}^{0}. \end{array}$$

We solve the system of equations to find $G_{14}^{0}$ and $G_{44}^{0}$, and as mentioned above, when considering the case in which the molecule is symmetric, this fact satisfies $G_{11}^{0}=G_{NN}^{0}$; that is, $G_{11}^{0}=G_{44}^{0}$, obtaining:

(A18) $$G_{14}^{0}=\frac{{\tilde{g_{s}}}^{2}{\tilde{g}}^{2}{\tilde{\tau }}^{2}\tilde{\upsilon }}{{\left(-1+\tilde{g_{s}}\tilde{g}{\tilde{\tau }}^{2}\right)}^{2}-{\tilde{g}}^{2}{\tilde{\upsilon }}^{2}}$$

and

(A19) $$G_{44}^{0}=\frac{\tilde{g_{s}}\left(1-\tilde{g_{s}}\tilde{g}{\tilde{\tau }}^{2}-{\tilde{g}}^{2}{\tilde{\upsilon }}^{2}\right)}{{\left(-1+\tilde{g_{s}}\tilde{g}{\tilde{\tau }}^{2}\right)}^{2}-{\tilde{g}}^{2}{\tilde{\upsilon }}^{2}}.$$

The Green’s functions that are obtained correspond to a molecular system with one thiophene ring. For molecular systems with two and three rings of thiophene, we proceed in a similar way, and the result of these calculations are shown below.

### Appendix A.2. Renormalization Process for Molecular Systems with Two Thiophene Rings

The system is determined by carbon and sulfur atoms with energy per site, $E_{C}$ and $E_{S}$, respectively, as shown in Figure A3a. They can be reorganized geometrically as shown in Figure A3b.

Because the rings have the same shape and the same components as the system with one thiophene ring, the result of the renormalization process is similar. Consequently, the effective local Green’s functions ($\tilde{g}$) of each site are also given by the expression (A8), and the effective coupling $\tilde{\upsilon }$ is given by the expression (A9) (see Figure A4).

In the same way, the relationships are fulfilled:

(A20) $$\tilde{\tau }=t_{\tau },\;\tilde{\delta }=t_{\delta }.$$

In this way, the molecular system with two thiophene rings (Figure A4) has been renormalized in a linear system, show in Figure A4, for which we must find the functions $G_{11}^{0}$, $G_{1N}^{0}$ and $G_{NN}^{0}$, which are determined by the Dyson equation.

As shown in Figure A4, in this case, there are six sites, so when calculating $G_{11}^{0}$, $G_{1N}^{0}$ and $G_{NN}^{0}$ with N=6, we get:

$$\begin{array}{cc} G_{16}^{0} & =\tilde{g_{s}}\tilde{\tau }G_{26}^{0},\\ G_{26}^{0} & =\tilde{g}\tilde{\upsilon }G_{36}^{0}+\tilde{g}\tilde{\tau }G_{16}^{0},\\ G_{36}^{0} & =\tilde{g}\tilde{\delta }G_{46}^{0}+\tilde{g}\tilde{\upsilon }G_{26}^{0},\\ G_{46}^{0} & =\tilde{g}\tilde{\upsilon }G_{56}^{0}+\tilde{g}\tilde{\delta }G_{36}^{0},\\ G_{56}^{0} & =\tilde{g}\tilde{\tau }G_{66}^{0}+\tilde{g}\tilde{\upsilon }G_{46}^{0},\\ G_{66}^{0} & =\tilde{g_{s}}+\tilde{g_{s}}\tilde{\tau }G_{56}^{0}. \end{array}$$

We solve the system of equations to find $G_{16}^{0}$ and $G_{66}^{0}$, and as mentioned above when considering the case in which the systems are symmetric, $G_{11}^{0}=G_{NN}^{0}$; that is, $G_{11}^{0}=G_{66}^{0}$, obtaining:

(A21) $$G_{16}^{0}=\frac{{\tilde{g_{s}}}^{2}{\tilde{g}}^{4}{\tilde{\tau }}^{2}{\tilde{\upsilon }}^{2}\tilde{\delta }}{{\left(-1+\tilde{g_{s}}\tilde{g}{\tilde{\tau }}^{2}+{\tilde{g}}^{2}{\tilde{\upsilon }}^{2}\right)}^{2}-{\tilde{g}}^{2}{\tilde{\delta }}^{2}{\left(-1+\tilde{g_{s}}\tilde{g}{\tilde{\tau }}^{2}\right)}^{2}},$$

and

(A22) $$G_{66}^{0}=\frac{\tilde{g_{s}}\left({\left(-1+{\tilde{g}}^{2}{\tilde{\upsilon }}^{2}\right)}^{2}-{\tilde{g}}^{2}{\tilde{\delta }}^{2}+\tilde{g_{s}}\tilde{g}{\tilde{\tau }}^{2}\left(-1+{\tilde{g}}^{2}\left({\tilde{\upsilon }}^{2}+{\tilde{\delta }}^{2}\right)\right)\right)}{{\left(-1+\tilde{g_{s}}\tilde{g}{\tilde{\tau }}^{2}+{\tilde{g}}^{2}{\tilde{\upsilon }}^{2}\right)}^{2}-{\tilde{g}}^{2}{\tilde{\delta }}^{2}{\left(-1+\tilde{g_{s}}\tilde{g}{\tilde{\tau }}^{2}\right)}^{2}}.$$

### Appendix A.3. Renormalization Process for Molecular Systems with Three Thiophene Rings

The system is determined by carbon and sulfur atoms with energy per site, $E_{C}$ and $E_{S},$ respectively, as shown in Figure A5a, and they can be reorganized, as shown in Figure A5b, by geometric visualization (because it does not affect the calculation of Green’s functions).

Because the rings have the same shape and the same components as the molecular system with one and two rings, the result of the renormalization process is similar. Therefore, the effective local Green’s functions ($\tilde{g}$) of each site are given by the expression (A8), and the effective coupling $\tilde{\upsilon }$ is given by the expression (A9), (see Figure A6).

In the same way, the relationships are:

(A23) $$\tilde{\tau }=t_{\tau },\;\tilde{\delta }=t_{\delta }.$$

In this way, the molecular system with three rings of thiophene (Figure A5a) has been renormalized in a linear system, as shown in Figure A6, for which we must find the functions $G_{11}^{0}$, $G_{1N}^{0}$ and $G_{NN}^{0}$, which are determined the Dyson equation. Figure A6 shows that, in this case, there are eight sites, therefore, when calculating $G_{11}^{0}$, $G_{1N}^{0}$ and $G_{NN}^{0}$ with N8, obtaining:

$$\begin{array}{cc} G_{18}^{0} & =\tilde{g_{s}}\tilde{\tau }G_{28}^{0},\\ G_{28}^{0} & =\tilde{g}\tilde{\upsilon }G_{38}^{0}+\tilde{g}\tilde{\tau }G_{18}^{0},\\ G_{38}^{0} & =\tilde{g}\tilde{\delta }G_{48}^{0}+\tilde{g}\tilde{\upsilon }G_{28}^{0},\\ G_{48}^{0} & =\tilde{g}\tilde{\upsilon }G_{58}^{0}+\tilde{g}\tilde{\delta }G_{38}^{0},\\ G_{58}^{0} & =\tilde{g}\tilde{\delta }G_{68}^{0}+\tilde{g}\tilde{\upsilon }G_{48}^{0},\\ G_{68}^{0} & =\tilde{g}\tilde{\upsilon }G_{78}^{0}+\tilde{g}\tilde{\delta }G_{58}^{0},\\ G_{78}^{0} & =\tilde{g}\tilde{\tau }G_{88}^{0}+\tilde{g}\tilde{\upsilon }G_{68}^{0},\\ G_{88}^{0} & =\tilde{g_{s}}+\tilde{g_{s}}\tilde{\tau }G_{78}^{0}. \end{array}$$

By solving the system of equations to find $G_{18}^{0}$ and $G_{88}^{0}$, when considering the case of a symmetric molecular system, where $G_{11}^{0}=G_{NN}^{0}$, that is, $G_{11}^{0}=G_{88}^{0}$, we obtain:

(A24) $$G_{18}^{0}=\frac{{\tilde{g_{s}}}^{2}{\tilde{g}}^{6}{\tilde{\tau }}^{2}{\tilde{\upsilon }}^{3}{\tilde{\delta }}^{2}}{A}$$

and

(A25) $$G_{88}^{0}=\frac{-\tilde{g_{s}}\left({\left(-1+{\tilde{g}}^{2}{\tilde{\upsilon }}^{2}\right)}^{2}\left(-1+\tilde{g_{s}}\tilde{g}{\tilde{\tau }}^{2}+{\tilde{g}}^{2}{\tilde{\upsilon }}^{2}\right)\right)}{A}+\frac{-\tilde{g_{s}}\left(\tilde{g^{2}}{\tilde{\delta }}^{2}\left(2-2\tilde{g_{s}}\tilde{g}{\tilde{\tau }}^{2}+{\tilde{g}}^{2}{\tilde{\upsilon }}^{2}\left(-2+\tilde{g_{s}}\tilde{g}{\tilde{\tau }}^{2}\right)\right)+{\tilde{g}}^{4}{\tilde{\delta }}^{4}\left(-1+\tilde{g_{s}}\tilde{g}{\tilde{\tau }}^{2}\right)\right)}{A},$$

where A is given by:

$$A=\left(1-{\tilde{g}}^{2}{\tilde{\upsilon }}^{2}\right){\left(-1+\tilde{g_{s}}\tilde{g}{\tilde{\tau }}^{2}+{\tilde{g}}^{2}{\tilde{\upsilon }}^{2}\right)}^{2}-2{\tilde{g}}^{2}{\tilde{\delta }}^{2}\left(-1+\tilde{g_{s}}\tilde{g}{\tilde{\tau }}^{2}\right){\left(-1+\tilde{g_{s}}\tilde{g}{\tilde{\tau }}^{2}+{\tilde{g}}^{2}{\tilde{\upsilon }}^{2}\right)}^{2}+{\tilde{g}}^{4}{\tilde{\delta }}^{4}{\left(-1+\tilde{g_{s}}\tilde{g}{\tilde{\tau }}^{2}\right)}^{2}.$$
